# Opioid Control for Patients With Sickle Cell Anemia in Emergency Departments in the Western Region of Saudi Arabia

**DOI:** 10.7759/cureus.69268

**Published:** 2024-09-12

**Authors:** Mansoor Radwi, Wisam Jamal, Abrar A Aljubayri, Abdulrahman S Hassan, Haitham S Alamri, Osama A Alraddadi, Saeed Alghamdi, Abdualrahman T Alashari, Hisham A Rizk

**Affiliations:** 1 Medicine, University of Jeddah, Jeddah, SAU; 2 General Surgery, Faculty of Medicine, University of Jeddah, Jeddah, SAU; 3 Medicine and Surgery, University of Jeddah, Jeddah, SAU; 4 Clinical Sciences, University of Jeddah, Jeddah, SAU; 5 Medicine and Surgery, Faculty of Medicine, Jeddah University, Jeddah, SAU; 6 Medicine, Faculty of Medicine, Jeddah University, Jeddah, SAU; 7 Faculty of Medicine, University of Jeddah, Jeddah, SAU; 8 Surgery, Faculty of Medicine, University of Jeddah, Jeddah, SAU

**Keywords:** adult sickle cell anemia, challenges in healthcare, emergency departments, opioid, treatment barriers

## Abstract

Introduction

Sickle cell anemia (SCA) affects the Saudi Arabian population significantly, with 4.2% carriers and 0.26% affected. Vaso-occlusive crises cause severe pain due to blood vessel blockage by sickled cells, leading to frequent emergency visits, where treatment includes the use of nonsteroidal anti-inflammatory drugs (NSAIDs), oxygen, and opioids. This study examines opioid use in managing SCA crises in Saudi emergency departments.

Method

A retrospective cross-sectional study in western Saudi Arabia surveyed board-certified emergency physicians (consultants, fellows, specialists) via the Sickle Cell Disease Implementation Consortium (SCDIC). Non-board-certified physicians and general practitioners were excluded. Data were gathered through structured surveys and supplemented by interviews.

Results

The study included 53 emergency physicians, mostly specialists (60.4%), with one to three years' experience, primarily from King Abdul Aziz University Hospital (28.3%) and King Abdullah Medical Complex (22.6%). While more than half of the participants felt adequately trained for SCA management, 20.8% faced challenges referring patients to case management programs. Major barriers included department overcrowding and the opioid epidemic.

Conclusion

The study reveals challenges in managing SCA in Saudi emergency departments, particularly with opioid use. Despite physician training, gaps in administrative support, medication access, and follow-up care persist. Institutional policies and opioid epidemic concerns restrict Schedule-II opioid prescriptions. Implementing a comprehensive opioid administration tracking system and standardizing protocols are crucial for enhancing patient outcomes. Future efforts should focus on improving resources and inter-institutional collaboration.

## Introduction

Sickle cell anemia (SCA) is a hereditary blood disorder that imposes a significant burden on millions worldwide. In Saudi Arabia, the carrier rate for the sickle cell trait is reported to be 4.2%, with 0.26% of the population being affected by sickle cell disease (SCD) [1]. Among the various complications of SCA, sickle cell crisis, or vaso-occlusive crisis (VOC), is particularly challenging, as it is characterized by painful episodes that occur due to the occlusion of blood vessels by sickled cells, prompting patients to seek relief in emergency departments (EDs). Depending on the severity of pain and other factors, patients may be admitted to hospitals or managed on an outpatient basis. Management protocols typically involve the administration of nonsteroidal anti-inflammatory drugs (NSAIDs), oxygen therapy, and opioids [2]. This study focuses on opioid usage in EDs to assist SCA patients in pain management.

Despite opioids being a cornerstone in the management of acute exacerbations of SCD, emergency physicians often struggle to address patients’ pain adequately due to the absence of definitive physical examination findings [3]. Moreover, the use of opioids in SCA management is hindered by prevailing stigma and concerns regarding addiction, compounded by the frequent and sequential dosing patterns observed in emergency settings. These apprehensions are exacerbated by the potential adverse effects of opioids, including excessive sedation, respiratory depression, addiction, and the development of tolerance to the drug [4].

This study aims to evaluate the efficacy of opioid use in EDs to assist pain management in SCA patients. It advocates for the implementation of a comprehensive program accessible across all EDs to track patients’ opioid administrations, including dosage and timing. This approach aims to empower physicians with pertinent data to facilitate informed decision-making regarding opioid administration and potentially prompt the consideration of alternative treatments to mitigate the risk of opioid-related complications while ensuring optimal pain management for patients with SCA [5].

## Materials and methods

Study design and period

This retrospective cross-sectional study included board-certified emergency physicians (consultants, fellows, and specialists) practicing in the western region of Saudi Arabia. The study was conducted from October 2023 to March 2024. This duration was determined by logistical considerations, participant availability, and data collection requirements, ensuring an adequate sample size and comprehensive data collection.

Inclusion and exclusion criteria

Board-certified emergency physicians (consultants, fellows, and specialists) practicing in the western region of Saudi Arabia were included in the study. Non-board-certified emergency physicians, emergency physicians not practicing in the western region of Saudi Arabia, and general practitioners in EDs were excluded from the study.

Data collection tool

A structured survey developed by the Sickle Cell Disease Implementation Consortium (SCDIC) was used to collect data for this study [6]. This validated survey tool is designed to gather comprehensive information on opioid management practices in the ED setting for patients with SCA.

Data collection technique

Data were collected through a combination of interviews and surveys. Participants were approached for interviews, during which they were provided with the survey link to complete electronically. Interviews were conducted to gain additional insights and clarification regarding opioid management practices in EDs.

Data entry and analysis

Data were initially recorded in Microsoft Excel (Microsoft Corp., Redmond, WA) and then analyzed using the Statistical Package for Social Sciences (SPSS) Version 25.0 (IBM Corp., Armonk, NY). Descriptive statistics were used, with categorical data presented as frequencies (n) and percentages (%), and numeric data presented as means and standard deviations (SD). For inferential statistics, chi-square and Fisher’s exact tests were used. A p-value of <0.05 was considered significant.

Ethical considerations

Ethical approval for this study was obtained from the Bioethics Committee for Scientific and Medical Research at the University of Jeddah (approval number UJ-REC-153). Informed consent was obtained from all the participants, ensuring their voluntary participation, confidentiality, and anonymity. The study adhered to the ethical guidelines outlined in the Declaration of Helsinki and other applicable regulations.

By following this methodology, the study aims to comprehensively evaluate opioid management practices followed by board-certified emergency physicians in the western region of Saudi Arabia, contributing valuable insights to improve patient care and outcomes.

## Results

Baseline characteristics of study participants

This study included a total of 53 participants. The majority of them were specialists (N= 32, 60.4%), followed by consultants (N= 19, 35.8%), and, lastly, fellows (N= 2, 3.8%). The period of clinical experience varied, with most participants having one to three years of experience; 18.9% had one year of experience, 17% had three years of experience, and 15.1% had two years of experience. Doctors working at King Abdulaziz University Hospital, Jeddah, constituted the largest group of respondents (28.3%), followed by those working at King Abdullah Medical Complex, Jeddah (22.6%), as shown in Table 1.

**Table 1:** Duration of clinical practice and current center of working (N= 53).

Variable	Subgroups	Frequency	Percentage
How many years have you been in clinical practice?	1	10	18.9
	2	8	15.1
	3	9	17.0
	4	2	3.8
	5	3	5.7
	7	4	7.5
	8	1	1.9
	9	3	5.7
	10	2	3.8
	12	3	5.7
	13	1	1.9
	14	1	1.9
	15	2	3.8
	20	3	5.7
	24	1	1.9
At what Center are you currently working?	King Fahad Armed Forces Hospital, Jeddah	3	5.7
	King Faisal Specialist Hospital, Jeddah	1	1.9
	King Abdullah Medical Complex, Jeddah	12	22.6
	King Fahad General Hospital, Jeddah	5	9.4
	King Abdulaziz University Hospital, Jeddah	15	28.3
	King Abdulaziz Medical City, Jeddah	9	17.0
	East Jeddah General Hospital, Jeddah	2	3.8
	Al Jedaani Hospital	2	3.8
	Others	4	7.5

Practice characteristics

As shown in Table 2, more than half of the doctors (54.7%) who participated in the study strongly agreed that they have the knowledge to provide care to patients with SCD. A slightly higher percentage (56.6%) strongly agreed that they have the training to deliver care to patients with SCD. An equal percentage (49.1%) agreed or strongly agreed that they have the administrative support required to treat patients with SCD. Additionally, 50.9% agreed that they have access to the necessary medications. Furthermore, 43.4% agreed that they have the ability to fix a follow-up appointment for their patients with a sickle cell specialist following discharge. A slightly lesser percentage (41.5%) agreed that they have the ability to fix such an appointment for their patients with a primary care provider. However, 20.8% disagreed on being able to refer patients to a case management program upon discharge. More than half (54.7%) of the participants agreed that they work in an ED with sufficient nurse staffing to provide good pain management to patients with SCD, while 67.9% agreed that physician/provider staffing is sufficient. Additionally, 58.5% agreed that nursing staff ratios allow the ED to provide safe care, and 54.7% agreed that these ratios allow the ED to provide high-quality care. Lastly, 41.5% agreed that patients’ underinsurance or lack of insurance does not affect their ability to provide good care. More than two-thirds (69.8%) agreed that the workflow in the ED is conducive to providing high-quality care for patients in sickle cell pain crises.

**Table 2:** Practice characteristics of participants (N= 53). ED, emergency department; SCD, sickle cell disease

Statement	Strongly disagree		Disagree		Agree		Strongly agree		Don’t know		Rather not provide	
	N	%	N	%	N	%	N	%	N	%	N	%
I have the knowledge to provide care to patients with SCD.	1	1.9	0	0	23	43.4	29	54.7	0	0	0	0
I have the training to deliver care to patients with SCD.	1	1.9	0	0	21	39.6	30	56.6	0	0	1	1.9
I have the administrative support I need to treat patients with SCD.	0	0	0	0	26	49.1	26	49.1	1	1.9	0	0
I have access to medications I need to treat patients with SCD.	0	00	1	1.9	27	50.9	23	43.4	0	0	2	3.8
I have access to medications I need to treat pain in individuals with SCD.	0	0	0	0	28	52.8	24	45.3	0	0	1	1.9
I am able to make a follow-up appointment with a sickle cell specialist following discharge.	4	7.5	4	7.5	23	43.4	16	30.2	5	9.4	1	1.9
I am able to make a follow-up appointment with a primary care provider following discharge.	4	7.5	8	15.1	22	41.5	11	20.8	7	13.2	1	1.9
I am able to refer patients to a case management program upon discharge.	2	3.8	11	20.8	13	24.5	13	24.5	13	24.5	1	1.9
I work in an ED with sufficient nurse staffing to provide good pain management to patients with SCD.	0	0	3	5.7	29	54.7	17	32.1	1	1.9	3	5.7
I work in an ED with sufficient physician/provider staffing to provide good pain management to patients with SCD.	0	0	0	0	36	67.9	16	30.2	0	0	1	1.9
Adequate physician and provider staffing is essential to ensure effective pain management for patients with SCD	0	0	1	1.9	36	67.9	16	30.2	0	0	0	0
Nursing staff ratios allow our ED to provide safe care.	1	1.9	5	9.4	31	58.5	15	28.3	1	1.9	0	0
Our nursing staffing allows our ED to provide high-quality care.	0	0	5	9.4	29	54.7	16	30.2	2	3.8	1	1.9
A lack of insurance or being underinsured does not affect my ability to provide good care.	0	0	5	9.4	22	41.5	17	32.1	7	13.2	2	3.8
The workflow in our ED is conducive to proving high-quality care for sickle cell pain crises.	0	0	3	5.7	37	69.8	11	20.8	2	3.8	0	0

Care provision and adherence to the National Heart, Lung, and Blood Institute (NHLBI) recommendations and opioid prescribing patterns

As shown in Table 3, 39.6% of the respondents mentioned that they do not prescribe Schedule-II medications (i.e., opioid analgesics) to patients who request them upon discharge, with 40% citing that they were not allowed to do so. Additionally, 75.5% stated that they had protocols for treating sickle cell pain in the ED, and 62.3% stated that they use individualized dosing protocols. The majority (79.2%) were aware of the Ministry of Health (MOH) recommendations for the treatment of VOC, and 77.4% mentioned that they rely on the patient’s medical history for the diagnosis of SCD. However, 83% mentioned that they do not have access to a system that provides a confirmed diagnosis of SCD if the patient was diagnosed at another center, and 84.9% stated that they could not contact the diagnosing doctor at another center to discuss the patient’s status. Of the participants, 66% agreed that having records of patient doses of opioids would affect their management plan and 81.1% said that they would use alternative drugs or lower the dosage of opioids if the patient had been recently administered opioids during a VOC. All participants (100%) believe that there is a need to implement a system that documents records of opioid dosing for SCD patients that is accessible by every center in the region.

**Table 3:** Care provision and adherence to NHLBI recommendations and opioid prescribing patterns (N= 53). ED, emergency department; NHLBI, National Heart, Lung, and Blood Institute; SCD, sickle cell disease; VOC, vaso-occlusive crisis

Statement	Answer	Frequency	Percentage
Upon discharge from the ED for sickle cell pain, do you prescribe Scheduled-II medications (i.e., opioid analgesics) to patients who request them?	Yes	9	17.0
	No	21	39.6
	I have never taken care of an SCD patient	1	1.9
	I have never been asked by an SCD patient for opioid analgesics for their pain management	2	3.8
If no what are the barriers to prescribing Scheduled-II medications (i.e., opioid analgesics) to patients who request them?	To be done by the hematologist or consultant	7	35.0
	Not allowed	8	40.0
	Patient factors\behavior	2	10.0
	Fear of dependence	1	5.0
	Opioid epidemic	1	5.0
	Not available	1	5.0
Does your ED have protocols for treating sickle cell pain?	Yes	40	75.5
	No	6	11.3
	I don’t know	5	9.4
	Prefer not to respond	2	3.8
Does your ED use individualized dosing protocols to treat sickle cell pain?	Yes	33	62.3
	No	9	17.0
	I don’t know	9	17.0
	Prefer not to respond	2	3.8
Are you aware of the MOH recommendations for the treatment of VOC?	Yes	42	79.2
	No	11	20.8
Do you depend on a patient’s past medical history for the diagnosis of SCD?	Yes	41	77.4
	No	12	22.6
If the patient was diagnosed at another center, do you have access to a system that provides you with the confirmed diagnosis of SCD?	Yes	7	13.2
	No	44	83.0
	Prefer not to respond	2	3.8
Can you contact the doctor who made the diagnosis in another center to discuss the patient’s status?	Yes	6	11.3
	No	45	84.9
	Prefer not to respond	2	3.8
If you have records of patient doses of opioids, does it affect your management plan?	Yes	35	66.0
	No	17	32.1
	Prefer not to respond	1	1.9
If the patient had a recent administration of opioids during VOC, do you use alternative drugs or lower the dosage of opioids?	Yes	43	81.1
	No	7	13.2
	Prefer not to respond	3	5.7
Do you think there is a need to implement a system that documents records of opioid dosing for SCD patients that is accessible by every center in the region?	Yes	53	100.0

Barriers to care

The participants reported that overcrowding was the most common barrier hindering their treatment of patients with SCD in the ED, followed by the opioid epidemic (43.4%). Administrative support and lack of medical equipment were the least reported causes (7.5% each).

Factors associated with awareness of the MOH recommendations for the treatment of VOC

As shown in Table 4, a significant difference was observed between centers in applying individualized dosing protocols to treat SCD pain (P < 0.05), while no difference was found between different centers in having ED protocols to treat SCD pain.

**Table 4:** Center differences in treating SCD pain and individualized dosing protocol. No significant association was found between the basic characteristics of participants and awareness of the MOH recommendations for the treatment of VOC (P > 0.05). ^1^Chi-squared test ED, emergency department; SCD, sickle cell disease

Name of the center	ED protocol to treat SCD pain					Individualized dosing protocol to treat SCD pain				
	Yes	No	Don’t know	No response	P-value^1^	Yes	No	Don’t know	No response	P-value^1^
King Fahad Armed Forces Hospital, Jeddah	1	0	0	2	0.34	0	0	1	2	0.001
King Faisal Specialist Hospital, Jeddah	1	0	0	0		1	0	0	0	
King Abdullah Medical Complex, Jeddah	9	2	1	0		5	4	3	0	
King Fahad General Hospital, Jeddah	5	0	0	0		5	0	0	0	
King Abdulalziz University Hospital, Jeddah	10	3	2	0		13	0	2	0	
King Abdulaziz Medical City, Jeddah	7	0	2	0		2	5	2	0	
East Jeddah General Hospital, Jeddah	1	1	0	0		2	0	0	0	
Al Jedaani Hospital	2	0	0	0		1	0	1	0	
Others	4	0	0	0		4	0	0	0	

## Discussion

The findings of this study underscore the complexities and challenges associated with the management of SCA, particularly in the context of EDs in the western region of Saudi Arabia. The findings reveal that while the majority of emergency physicians possess the requisite knowledge and training to provide care to individuals with SCD, there are significant gaps in administrative support, access to medications, and the ability to facilitate follow-up appointments with specialists or primary care providers [7]. These gaps may contribute to the suboptimal management of SCD patients during VOCs, potentially leading to increased morbidity and healthcare use [8].

The reluctance to prescribe Schedule-II opioid analgesics upon discharge, cited by nearly 40% of the respondents as a barrier due to institutional policies or fear of contributing to the opioid epidemic, highlights the tension between the need for effective pain management and concerns over opioid misuse. This tension is further exacerbated by the lack of a unified system to track opioid dosing across different healthcare centers, which could help in making informed decisions regarding pain management [9,10].

More than half of the respondents agreed on the need for individualized dosing protocols for treating SCD pain, yet only 62.3% reported using such protocols. This suggests a discrepancy between the recognition of best practices and their implementation. This discrepancy may be due to various factors, including the lack of resources, insufficient training, or other administrative barriers [11,12].

The significant difference observed in the application of individualized dosing protocols across different centers underscores the variability in the quality of care provided to SCD patients. This variability may be due to differences in institutional policies, the availability of resources, or the level of expertise among healthcare providers [13].

The lack of significant association between the basic characteristics of participants and awareness of MOH recommendations for the treatment of VOCs indicates that awareness of these guidelines is widespread, regardless of clinical experience or position. However, the gap between awareness and adherence to these recommendations in practice suggests a need for targeted interventions to improve guideline implementation. The study’s limitations include its reliance on self-reported data, which may be subject to recall bias or social desirability bias. Additionally, the study’s focus on emergency physicians practicing in the western region of Saudi Arabia may limit the generalizability of its findings to other regions or healthcare settings. Other limitations include a small sample size and the retrospective nature of the study, which may affect the robustness of the results.

## Conclusions

This study highlights the significant challenges faced by emergency physicians in managing SCA in the western region of Saudi Arabia, particularly concerning opioid use. While physicians possess the necessary knowledge to treat pain crises, gaps in administrative support, access to medications, and follow-up care persist. The reluctance to prescribe Schedule-II opioids due to institutional policies underscores the need for a balanced approach to pain management. Future efforts should focus on standardizing treatment protocols and improving resource availability to enhance patient care.
